# Comparing the old to the new: A comparison of similarities and differences of the accreditation standards of the chiropractic council on education-international from 2010 to 2016

**DOI:** 10.1186/s12998-018-0196-9

**Published:** 2018-08-15

**Authors:** Stanley I. Innes, Charlotte Leboeuf-Yde, Bruce F. Walker

**Affiliations:** 1 School of Health Professions, Murdoch University, Murdoch, Australia; 2 Institut Franco-Européen de Chiropraxie, Ivry sur Seine, France; 3 CIAMS, Université Paris-Sud, Université Paris-Saclay, 91405 Orsay Cedex, France; 4 CIAMS, Université d’Orléans, 45067 Orléans, France; 5 Institute for Regional Health Research, University of Southern Denmark, DK-5000 Odense, Denmark

**Keywords:** Accreditation, Critical review, Chiropractic, Education, Standards

## Abstract

**Background:**

Chiropractic programs are accredited and monitored by regional Councils on Chiropractic Education (CCE). The CCE-International has historically been a federation of regional CCEs charged with harmonising world standards to produce quality chiropractic educational programs. The standards for accreditation periodically undergo revision. We conducted a comparison of the CCE-International 2016 Accreditation Standards with the previous version, looking for similarities and differences, expecting to see some improvements.

**Method:**

The CCE-International current (2016) and previous versions (2010) were located and downloaded. Word counts were conducted for words thought to reflect content and differences between standards. These were tabulated to identify similarities and differences. Interpretation was made independently followed by discussion between two researchers.

**Results:**

The 2016 standards were nearly 3 times larger than the previous standards. The 2016 standards were created by mapping and selection of common themes from member CCEs’ accreditation standards and not through an evidence-based approach to the development and trialling of accreditation standards before implementation. In 2010 chiropractors were expected to provide attention to the relationship between the structural and neurological aspects of the body in health and disease. In 2016 they should manage mechanical disorders of the musculoskeletal system. Many similarities between the old and the new standards were found. Additions in 2016 included a hybrid model of accreditation founded on outcomes-based assessment of education and quality improvement. Both include comprehensive competencies for a broader role in public health. Omissions included minimal faculty qualifications and the requirement that students should be able to critically appraise scientific and clinical knowledge. Another omission was the requirement for chiropractic programs to be part of a not-for-profit educational entity. There was no mention of evidence-based practice in either standards but the word ‘evidence-informed’ appeared once in the 2016 standards.

**Conclusions:**

Some positive changes have taken place, such as having bravely moved towards the musculoskeletal model, but on the negative side, the requirement to produce graduates skilled at dealing with scientific texts has been removed. A more robust development approach including better transparency is needed before implementation of CCE standards and evidence-based concepts should be integrated in the programs. The CCE-International should consider the creation of a recognition of excellence in educational programs and not merely propose minimal standards.

## Background

Governments encourage, directly and indirectly, a range of strategies to regulate, monitor and improve the organization, management, quality, and safety of health services. One of these strategies is the accreditation of health training programs, which are now employed in over 70 countries [1, 2]. Accreditation is perceived to be one lever to stimulate systems-level improvement by promoting uptake of optimal, evidence-based governance and clinical standards [3].

Following this model, the role of the Councils on Chiropractic Education (CCEs) is to oversee the regulatory standards of chiropractic education worldwide. Such CCEs are found in Australia (CCE-Australasia), Canada (CCE-Canada), Europe (European-CCE), and in the USA (CCE-USA).

In 2003 an international umbrella council, known as the Council on Chiropractic International (CCE-International) was established as a federation consisting of representatives from the four previously mentioned CCEs. The various CCEs developed a list of the minimum expectations for standards that they could agree upon. In part, this was influenced by the differing types of authorization that some CCEs were themselves subject to. This list was adopted as the Accreditation Standards for the CCE-International, with the intent of harmonising world standards for excellence in chiropractic educational programs [4]. The CCE-International is importantly and strategically placed to guide chiropractic education, given it is recognised by the World Health Organisation as the source of information regarding the evaluation of chiropractic education [5]. The CCE-International is not an accrediting agency per se. Rather, the CCE-International has historically provided guidance and support for its four members and others wishing to join the CCE-International on appropriate educational standards and accreditation processes for the achievement of high quality education by chiropractic programs. Presently however, it appears that the CCE-USA has withdrawn from this collaboration as it is no longer listed as a member of the CCE-International on its website and is recorded as being a CCE-International member agency in the 2016 Glossary section between 2001 and 2016 [6]. We could find no official statement from the CCE-International on this change nor did we have a response to a written enquiry confirming the change and any attendant reasons for it. A written enquiry was sent to the CCE-USA seeking their reasons for withdrawal from the CCE-international. The CCE-USA declined to respond as the notification and reasons for termination of membership was communicated to the CCE-International and as such was a confidential communication between two parties. Consequently disclosure was at the discretion of the CCE-International (Email to CCE-USA (cce@cce-usa.org) January 2018).

The CCE-International standards and processes generally consist of several components including among other things an expectation for adequate physical resources, such as buildings, staff and finances. Also defined is a set of competencies a student should acquire before graduation. Regulatory agencies expect that the program curriculum will be designed to achieve a specified set of knowledge proficiencies, skills and abilities. This aims at guaranteeing that chiropractors achieve a similar basic standard, regardless where in the world they obtain their education. The attainment of the set competencies and standards is intended to ultimately improve the quality of societal levels of health care and patient safety. Finally, these standards define the processes for initial accreditation as well as re-accreditation with the aim of providing a process that leads to continual improvement of the program.

Juxtaposed against these ideals is the reality of elements of undesirable chiropractic standards of practice in the wider community documented over the past ten years, where it has been argued that this conduct may be associated with variations between chiropractic programs [7–9]. These undesirable practices include negative vaccination beliefs, excessive X-ray usage, non-evidence-based treatment choices and the infrequent referral to or from other health care providers [7, 9]. These undesirable activities have been described as being in contrast to current scientific paradigms, such as evidence-based practice, and aligning with scientifically unorthodox/subluxation or vitalist model [7]. It is not unreasonable to expect that CCE requirements would therefore include elements that counteract the teaching of undesirable practice patterns, such as a non-evidence approaches to care. However, this may not be the case as recent studies have compared international chiropractic accreditation standards and graduate entry-level competencies and found considerable variation between them [8, 10, 11].

Government agencies frequently engage in the development and revision of accreditation standards [12]. It is logical to assume that such revisions are intended to improve the standards and processes with the ultimate outcome of improving graduate abilities and thus public health and safety. It is also logical to assume that revisions are based on responses to practice patterns, both desirable and undesirable, and their trends in different parts of the world. The acquisition of such information from a variety of stakeholders is widely recognised as being a foundational component for the construction of accreditation standards to ensure they are socially responsible [13].

Therefore, one would expect to see a positive incremental change in these domains over time. To date this type of change has not been studied for CCEs. Instead of investigating such changes in each CCE on its own, it is appropriate to scrutinize the CCE-International, as it is expected to broadly reflect the CCE standards world-wide. In addition, it is independent of any regulatory authority and is responsible for the development of its own standards, thus truly reflecting leadership and the intentions of the educational community within the chiropractic profession.

### Objectives

The objectives of this review were (i) to compare the Council on Chiropractic Education International 2016 Accreditation Standards with their previous 2010 Accreditation Standards, including the way they were developed, and (ii) to explore similarities and differences of prescribed recommendations to identify any changes to procedures, concepts and emphases. And, finally, (iii) to comment on whether these changes are likely to be for the better or the worse.

## Methods

We conducted a systematic investigation into the first two objectives. This initially involved a critical look at the development process, followed by a comparison of the themes covered in the CCE-International Accreditation Standards from 2010 and 2016. This was followed by a comparison of the content of the two documents looking for similarities and differences. As part of the analysis we counted pre-selected key words and compared them for increased or decreased frequency of usage. We were particularly interested in how the topic of evidence-based teaching would be covered and how an evidence-friendly culture would be developed, as an important aspect of modern health-care education and delivery.

### Data extraction process and synthesis of results

The CCE-International website was searched for the current Accreditation and Educational Standards.

The current CCE-International Framework for Chiropractic Education and Accreditation was downloaded in March 2017 [4] and the publication approval date was identified as June 2016. The publication date of the previous CCE-International standards could not be determined from the CCE-International website. An email in April 2017 was sent via the CCE-International server requesting this information. No response was received. A web library [14] was used to search CCE-International website history to find information about the date for the previous standards, which was found to be November 2010. This matched information used in a prior study [15].

The PDF texts of the downloaded 2010 and 2016 CCE-International standards were converted to Microsoft Word format. The Word documents were compared to the PDF texts to ensure that no errors had occurred. The 2016 standards were structured into 4 sections (themes): Introduction/Foreword, Standards, Competencies, and Processes.

Accordingly, we divided all information from the 2010 standards into individual components and then arranged them to match the four sections of the 2016 standards. This allowed for direct comparison of similarities and differences in a Microsoft Word document.

A comparison of contents was made by counting words in the two documents. The “Glossary” section of the 2016 standards was not included in the word count as there was no equivalent section in the 2010 standards and it only contained definitions of words and the rationale for their use. Content analysis using word counting is widely used in qualitative research [16–18]. A summative content analysis involves reading the data several times for familiarisation to provide the opportunity to reflect on the overall meaning. The data was then coded and compared, usually for keywords or content and generally tabulated [18]. This process was to facilitate the subsequent interpretation of the underlying context. After this process, the lead researcher identified sixty-seven predominately adjectival words, seen in Table 3, considered to reflect the content and intent of the educational standards. These words related to the administration, teaching, or practice of chiropractic as well as the assessment of a chiropractic program (CP). The lead author (SI) then searched for each word using the ‘Find’ function in Microsoft Word. All occurrences of the word were copied verbatim, including the sentence in which it was found so it could be seen in its context, and listed in a spreadsheet. These final list was reviewed and discussed with another of the authors (CLY).

The second phase of the investigation determined the frequency of the use of each word and whether it was being directed toward the student, the CP, was a heading (in larger font or bold indicating a section of information) or if it had another unrelated purpose. The context or intent of the use was then determined by following the categorization of ‘heading’, ‘student’, ‘CP’ or ‘other’. For example, the word ‘respect’ was searched for in the 2016 standards. It occurred as an expectation that a student would “respect the cultural diversity of patients” (classified as ‘student’), and that the accreditation process would “respect the autonomy of the CP” (classified as ‘CP’), and that there was a need to “meet CP objectives with respect to student criteria” and therefore classified as ‘other’. Thereafter the frequency of use was established for each word and category. Uncertainty over the intent of any word was discussed with the second author (CLY). Any disagreement between the two authors was resolved by discussion with the third author (BW).

In the final phase, the extracted spreadsheet was visually examined for an increased, decreased or unchanged frequency of the occurrence of the words when compared across CCE-International standards for 2010 and 2016.

## Results

There was a high degree of agreement between the two researchers on the classification of the similarities and differences and the context of the prescribed key words. The third researcher was therefore not required to resolve any disagreements.

### General impressions

The documents contained the same number of sections although these were labelled differently. In general, more descriptive detail was added to each section in the newer version, making the 2016 CCE-International Accreditation Standards 2.7 times larger than the 2010 standards, (7042 words versus 2280 words, respectively not including Foreword/Introduction sections).

The four sections were:

1. Foreword (2010 standards) 421 words, Introduction (2016 standards) 701 words.
2. Educational Standards (2010) 468 words, Program Standards (2016) 2005 words.
3. Educational Objectives (2010) 471 words, Competencies for Graduating Chiropractors (2016) 1540 words.
4. Process of Accreditation (2010) 799 words, Accreditation Policies and Procedures (2016) 3341 words. This large difference is due to the 2016 standards containing an additional section for Reaffirmation of Accreditation (1656 words).

Both standards provide a definition of chiropractic (Table 1). The 2016 accreditation standards have adopted the definition of the World Federation of Chiropractic (WFC) [19] whereas the 2010 standards’ definition is unreferenced. The adoption of the WFC definition has resulted in a narrowing of the scope from “giving particular attention to the relationship of the structural and neurological aspects of the body in health and disease” to “the diagnosis, treatment and prevention of mechanical disorders of the musculoskeletal system”.

**Table 1:** Comparison of definitions of chiropractor/chiropractic used in the 2010 and 2016 Council on Chiropractic Education – International Accreditation Standards

Standards	Definition of Chiropractor
2010	The chiropractor, as a practitioner of the healing arts, is concerned with the health needs of the public. He/she gives particular attention to the relationship of the structural and neurological aspects of the body in health and disease; he/she is educated in the basic and clinical sciences as well as in related health subjects. The purpose of his/her professional education is to prepare the chiropractor as a primary health care provider. As a portal of entry to the health delivery system, the chiropractor must be well educated to diagnose, to care for the human body in health and disease and to consult with, or refer to, other health care providers when appropriate for the best interest of the patient. (Pg 1)
2016	‘A health profession concerned with the diagnosis, treatment and prevention of mechanical disorders of the musculoskeletal system, and the effects of these disorders on the function of the nervous system and general health. There is an emphasis on manual treatments including spinal adjustment and other joint and soft-tissue manipulation.’ (Pg 17)

### Method of development of new standards

According to the 2016 Standards’ ‘Development process’ section, the standards were initially developed by a mapping of common themes with a computer software qualitative research program (NVivo) of the 4 member CCEs. In 2014 and 2015 a Steering Committee with representatives from each of the four member agencies met to critically review the draft framework. The members of the Steering Committee are named along with their membership affiliations but their qualifications and expertise to perform this task are not. The CCE-International Board was said to have approved progress at a number of ‘key stages’ throughout this process. In April 2015, the draft framework went through a consultation process with the four CCE-International member agencies (participants and qualifications not named). A second round occurred in November 2015. This was described as being with stakeholders ‘more broadly’, but the identity of these stakeholders, their qualifications, or expertise is not described. At each stage feedback was ‘considered’ and incorporated by the Steering Committee and a final decision of approval was made by the CCE-International in June 2016.

No information could be found regarding the conception of the 2010 CCE-International Standards.

### Foreword section of the accreditation standards in 2010 versus 2016

*Similarities:* These CCE-International standards are stated to constitute a minimum requirement for chiropractic program (CP) accreditation. Any CCE seeking membership to the CCE-International is expected to adopt and meet these standards.

Both 2010 and 2016 versions recognise the need to accommodate cultural and regional differences (Table 2).

**Table 2:** Comparison of CCE-International Accreditation / Educational standards 2010 and 2016

Domain and subdomain	2010	2016
Introduction/Foreword		
Definition of Chiropractor	Self-defined	Use of the definition by the World Federation Chiropractic
Areas must address	X	X
Recognition of cultural variations	X	X
Intention to be used as reference	X	X
This is a minimum standard	X	X
1. PROGRAM STANDARDS		
Based on model of outcomes-based education		X
CCE must monitor exit outcomes		X
Exit outcomes must be explicit		X
Must be communicated to all stakeholders		X
Curriculum must achieve educational outcomes		X
Monitor & evaluate curriculum effectiveness	X	X
Goals		X
Must define its mission, measurable goals & objectives		X
Mission must incorporate		X
Instruction / learning		X
Patient care		X
Research & scholarship		X
Service		X
Participation-consult with principal stakeholders		X
Autonomy to develop own program		X
Ethics, integrity & accountability	X	X
Governance		X
Governing board		X
Governing structures		X
Academic leadership		X
Faculty participation		X
Student input		X
Administration		X
Evaluation & quality improvement		X
Patient care		X
Educational budget & resource allocation	X	X
Educational Program	X	X
Curriculum model & educational methods	X	X
Curriculum development & assessment		X
Curriculum structure & content	X	X
Faculty		
Minimal Qualifications		X
Students	X	X
Student admissions		X
Disclosure to students		X
Student support services		X
Student policies	X	X
Student competencies	X	X
Assessment of student performance	X	X
Research & Scholarship	X	X
Resources	X	X
Physical facilities	X	X
Clinic resources	X	X
Learning resources	X	X
Information and communication technology		X
Service		X
2. COMPETENCIES		
Definition Competence	X	X
Definition of Standard	X	X
Foundational knowledge	X	X
Clinical skills	X	X
Formulate a differential diagnosis	X	X
Develop & evolve a management plan		X
Implement & monitor treatment		X
Evaluation of progress		X
Professionalism		X
Ethics & jurisprudence		X
Record keeping		X
Communication skills	X	X
Chiropractor-patient relationship	X	X
Inter-professional collaboration	X	X
Health Promotion & disease prevention	X	X
3. PROCEDURES		X
Initial Accreditation		X
Reaffirmation of accreditation		X
Confidentiality		X
1. Initial application for accreditation		X
Letter of intent		X
Eligibility criteria	X	X
Evidence of eligibility	X	X
Self-evaluation report (SER)	X	X
Decision about SER	X	X
Site team visit	X	X
Joint activities in accreditation process		X
Site team report		X
Final decision to ward accredited status		X
Award of Accredited status		X
Deferral of accreditation		X
Denial of accreditation		X
Notification of decision		X
2. Reaffirmation of accreditation		X
Letter of intent		X
Eligibility criteria		X
SER		X
CCE decision on SER satisfactory / unsatisfactory		X
Site team visit		X
Joint activities in accreditation process		X
Site team report		X
Final decision to ward accredited status		X
Award of Accredited status		X
Deferral of accreditation		X
Impose sanctions		X
Refusal to reaffirm		X
Notification of decision		X
Reaccreditation-reinstatement following refusal		X
Status description		X
Monitoring	X	X
Reports	X	X
Special actions		X
Quality assurance of the CCE for its improvement		X
Complaints and appeals		X
Role of Governance structure of the CCEI member		X
Not included in the 2016 from 2010		
appreciates chiropractic history and the unique paradigm of chiropractic health care	X	
acquires the ability critically to appraise scientific and clinical knowledge	X	
select research subjects, design simple research methods, critically appraise clinical studies and participate in multi-disciplinary research programs	X	
accept the responsibilities of a chiropractor	X	

*Differences:* None were found.

### Program standards (educational standards in 2010 versus 2016)

*Similarities:* The 2016 and 2010 standards share domains that address student policies, competencies, and assessment of performance and the educational program. Also shared are the requirements for adequate physical facilities, faculty, support staff, research, scholarship, clinic and learning resources. CPs are expected to be ethical and their advertising and marketing should reflect integrity in all matters. In addition, the program standards should meet local judicial and legal requirements.

Other commonalities are that mission, objectives and goals should be clearly stated for each CP. There must be financial transparency and enough resources in order for the most recently enrolled students to be able to graduate. The appropriate overseeing governing body of the CP should be allowed to act with autonomy. Input is expected from faculty, staff, students, patients, and appropriate others. Finally, there should be logic and structure to the curriculum that must be scaffolded with appropriate pedagogy and resources in order to achieve the CP objectives.

*Differences:*

Added:

The 2016 standards have moved to a hybrid model of outcomes-based education alongside self-assessed quality improvement. This means that each CP must provide an educational environment and curriculum as well as monitor and evaluate the effective acquisition of the knowledge, skills and attitudes needed to achieve the exit outcomes as described by competencies for graduating chiropractors. These must be clearly communicated to all concerned. Considerably more detail than before is provided for appropriate Governance and Administration.

The CP must regularly publish an academic calendar/catalogue, bulletin or similar document. This document should contain information for current and potential students that is accurate and relevant.

Other additions include standards for ‘information and communication technology’ and service to the program. The 2016 standards contain the additional expectation that patient care should be “evidence-informed” and should incorporate quality assurance. There was no mention of evidence-based care at all in the 2010 document nor was it explicitly mentioned in the 2016 version.

Staffx/faculty must be engaged in research and scholarship, service, professional development and governance activities as well as undergo regular performance reviews. The planning, goals and objectives of research should support the CP mission and facilitate the relationship between teaching and research. Faculty should be qualified by virtue of their academic and professional training and experience and/or their credentials to be educators.

Omitted:

Removals from the 2016 standards include the requirement for stable academic staff and that clinical staff should have as a minimum 3 years fulltime practice or 2 years teaching experience and be registered. CPs are no longer required to operate as, or as part of, an institute established as a not-for-profit educational entity.

### Competencies/educational objectives

*Similarities:* Shared are the standards for a competent clinical encounter with a patient; a foundational knowledge, clinical skills as evidenced by the ability to formulate a diagnosis, implement treatment whilst demonstrating communication skills, a quality chiropractor-patient relationship, professionalism, and inter-professional collaboration.

*Differences:*

Added

The focus of the standards has remained on chiropractors serving as primary contact practitioners and a portal of entry into health care but the standards now also include the need to perform tasks safely and effectively in a specific workforce setting.

The clinical skills domain has been expanded to include the need for a developed management plan and its monitoring as well as appropriate informed consent which includes treatment risks, benefits, natural history and alternative treatment options.

A domain for inter-professional collaboration has been added along with the need to be able to recognize the limits of individual and professional knowledge and competence.

There are now also expectations for inclusion of psychosocial factors in patient assessment and interventions. The appropriate and effective delivery of care has been expanded to include interventions other than spinal manipulation. Finally, chiropractors are expected to become active participants in health promotion and disease prevention for the communities and societies they serve.

Omitted

The 2016 standards do not include the 2010 requirements for the graduate to “appreciate chiropractic history and the unique paradigm of chiropractic health care”. Additionally, students are not required to be able to select research subjects, design simple research methods, critically appraise scientific and clinical knowledge, and participate in multi-disciplinary studies. Finally, removed is also the requirement that graduates achieve a level of skill and expertise in manual procedures emphasizing spinal manipulation, regarded as “imperative within the chiropractic field”.

### Procedures for initial accreditation and reaccreditation

*Similarities:* The 2010 CCE-International accreditation standards primarily focus on initial accreditation. The standards for reaccreditation are stated as being the same as for accreditation and are regarded in that manner for comparative purposes with the 2016 standards.

Both standards expect the accreditation to begin with notification by the program to the CCE of intent to pursue accreditation. It is expected that the program will have met the specified eligibility criteria stated in the accreditation/education standards.

Once eligibility is established, both standards expect the production of a self-evaluation report. The CCE is empowered to ask questions that may arise from the self-evaluation report. If this report is deemed satisfactory, then a site inspection is conducted to determine agreement between the report and expected accreditation standards. CPs are to be given the opportunity to address errors of fact before the inspection team report is submitted, as well an opportunity to respond to the final report.

Both standards require the availability of appeal processes for decisions made by the member CCE. The CCE options at the end of the process to award, defer or deny accreditation remain.

*Differences*

Added:

There should be transparent communication between the CCE and the CP.

All aspects of the accreditation process should be confidential, such as the self-evaluation report, inspection team reports, and the final report and recommendations. All documentation and the self-evaluation report remain the property of CP. This right is waived if the CP publishes any of the accreditation documentation.

The 2016 standards gain an expectation that the inspection site team members should be qualified, although these required qualifications are not specified. CPs have the right to object to the inclusion of a particular inspection team member, if there is a conflict of interest (not specifically defined in the 2016 standard). The site inspection team can fully evaluate all aspects of the program at a mutually convenient time.

In the re-accreditation process, there is an additional option available to CCEs to impose sanctions, although these are not specified. Other variations include the keeping of an up-dated list of accredited programmes on the member CCE website, details for the regular monitoring of programs, and special actions for extraordinary circumstances. Finally, the member CCEs are expected to make themselves available for feedback at the end of the process for quality assurance and continued improvement.

The deferral option, when a CCE requires additional information in order to make a final decision to reaffirm accreditation, is now considered to be confidential. Public notification is required once the decision to award or deny accreditation has been made.

The notification of the final decision regarding (re)accreditation should be provided within 30 days of the final meeting of the CCE and the CP. The CCE is required to publish and maintain the date of the initial accreditation and the length of time it was awarded for on the CCE website. This should also include the year of the next comprehensive site visit. The CCE is expected to keep the decision to impose sanctions confidential and not release this information to the public. There is no requirement for the CCE to publish the reasons why accreditation or reaccreditation was accepted or refused or the strengths or weaknesses of the CP as gathered from the inspection process.

Omitted:

Previously, notification of the accreditation decision should be given to the CP within 90 days, compared to 30 days in the new version.

### Word analysis/frequencies (Table 3)

The 2016 CCE-International standards are approximately 3 times larger than the 2010 standards. Consequently, we decided that, at a minimum, a key word should be at least 3 times more or less frequent to warrant inclusion in this section of the analysis. As compared to ‘0’ in the 2010 standards, any positive mention of a new keyword in the 2016 text would be considered relevant.

**Table 3:** The frequency of key words (or their derivatives) in the 2010 and 2016 CCE-International Accreditation Standards

	2010 Standards					2016 Standards				
Word	Total number	Headings	Student	CP	Other	Total Number	Headings	Student	CP	Other
Accountability	1			1		2	1		1	
Accredit	40	14		26		59	10			49
Assessment	2			1	1	13	2	7	4	
Attitudes	1		1			3		3		
Autonomy	0					5	1		4	
Care	18		6		12	36	1	10	4	21
Chiropractic/or	48					191				
Clinical	16		8		8	15	2	13		
Collaboration	0					5	2	3		
Communicate	2		2			12	3	4	5	
Competent	8		8			53	2	39		12 in footnotes
Compliance	1			1		14			14	
Confidentiality	0					2	1		1	
Consult	3		3			2		1	1	
Contra-indication	0					0				
Criteria	0					5	2		3	
Curriculum	8	1		7		20	3		17	
Define	3			3		3	1		1	1 footnotes
Development	7	1		6		7	1	1	4	1
Diagnose	6		6			7	1	6		
Disease	3		3			7	2	5		
Disclosure	0					2	1		1	
Effective	4		2	2		16		6	10	
Engage	0					3		1	2	
Ethic	2		1	1		10	2		8	
Evaluate	12	2		10		32	4		28	
Evidence	5			5		24		1	23	
Facilitates	1		1			2		2		
Faculty	1					14	2		12	
Goal (s)	3	1		2		19	2	1	16	
Identify	2			2		16		7	9	
Indicate	2			2		3		2		
Improvement	1			1		8	1	2	5	
Integrity	0					7	1		6	
Interprets	4		2	2		3		3		
Inter-professional	0					2	2			
Knowledge	11		7	4		14	2	12		
Leadership	0					11			11	
Limit	0					3		1	2	
Measure	0					1		1		
Method	3		1	2		8	1	6		
NeuroMSK	4		4			3		3		
Outcome	1	1				22	2			5 footnotes
Patient	7		7			34	3		31	
Participation	2		1	1		5	2		3	
Perform	3		2	1		11	1	7	3	
Policies	7	2		5		22	3		19	
Prevent	0					9	2	7		
Promotion	2			2		9	2	4	3	
Public	4		1	3		8		1	7	
Recognize	3				3	6		5	1	
Research	10	1	4	5		13	2		11	
Resources	7	4		3		13	3	9		1 footnote
Respect	4		1			7		4	3	
Requirements	12			12		15			15	
Relationship	2		2			6	2	3	1	
Safe	2		1	1		6		5		1 footnote
Scholarship	1			1		5	1	4		
Scope of Practice	2		1	1		2		1		
Serve	1		1			4		4		
Skills	6		6			13	4	9		
Staff	11	1		10		5			5	
Stakeholder	0					8			8	
Standard	25	3		22		44	2		42	
Strategies	0					3		2	1	
Student	16			16		43	8		35	
Support	2			2		11		3	8	
Transparent	0					3			3	
Wellness	1		1			2		2		

Words that indicated a more integrated role for chiropractors in the health care system in the 2016 standards were ‘collaboration’ (0 in 2010 standards and 5 in the 2016 standards), ‘inter-professional’ (0 vs. 2), ‘serve’ (1 vs. 4) and ‘stakeholders’ (0 vs. 8).

Increased number of words indicating an awareness of a broader role for chiropractors was found for ‘prevention’ (0 vs. 9) and ‘promoting’ health (2 vs. 9).

Words that suggest a more outcomes-based approach to accreditation of CPs were ‘outcomes’ (1 vs. 22), ‘performance’ (3 vs.11), ‘evaluate/ing’ (12 vs. 32), ‘evidence’ (5 vs. 24), ‘goals’ (3 vs. 19), ‘effective’ (4 vs. 16) and ‘compliance’ (1 vs. 14).

There appears to be an adoption of more descriptive language for the standards for graduate competencies ‘communication/ing’ (2 vs. 12), ‘competence/tent’ (8 vs. 53), ‘integrity’ (0 vs. 7), ‘ethics’ (2 vs. 10), ‘engages’ (0 vs. 4), ‘leadership’ (0 vs. 11), ‘safety’ (2 vs. 6) and ‘scholarship’ (1 vs. 5). Additionally, in the 2016 standards an increase in the word ‘patient’ (7 vs. 34) may suggest they are more patient focused.

## Discussion

### Summary of findings

This is the first study to explore changes in CCE accreditation standards over time for indicators of progressive change.

The new and previous standards are similar in that they share the same broad framework for (re)-accreditation, adequate physical structure and staff to reach the CPs mission statement and objectives. They also share the expectation for the attainment of specific competencies that lead to the graduation of a competent chiropractor.

The new standards have provided more descriptive information of all the areas of accreditation and adopted a more contemporary hybrid model of accreditation, combining both outcome-based assessments and quality improvement of the CP [20]. This de-emphasises the structures and staff where chiropractic education takes place and moves toward expecting the CP to provide outcome measures that demonstrate the student is acquiring the skills, knowledge, and attitudes to become a competent chiropractor who safely and effectively delivers patient care. This process is intertwined with the expectation that this will lead to continuous quality improvement of the CP.

We found that CCE-International accreditation standards of 2016 have, in general, moved in a positive direction. However, some differences and omissions were not positive. These were not in accord with the evolution of public health frameworks that has seen a move toward engaging a broader range of stakeholders and a move toward the community collective values of transparency, evidence-based effectiveness, and accountability [21].

### Discussion of findings

#### Construction of accreditation standards

The current CCE-International standards were developed using a review process that was limited to its member agencies and ‘stakeholder’ consultations. The ‘stakeholders’ were not identified. Concerns have been raised about the lack of transparency for initiatives and changes being adopted by accreditation agencies [22–24]. Consequently to avoid the accusation of political bias or agenda, the qualifications, experience and affiliations of all participants and ‘stakeholders’ should be carefully selected and clearly stated. In addition, external health science educators outside of chiropractic and health consumer representatives should be involved.

High quality accreditation standards should involve a review of the evidence base for each standard, new material development, and a field methodology to trial and refine the new standards [11, 22].There was no information on whether this involved a comprehensive review of the evidence base for each standard, nor was a field trial reported to have been conducted. Past research has already raised questions about the absence of an evidence-based approach in CCE accreditation standards and this lack of rigour raises further concerns over their validity [9, 10, 13].

Perhaps reflective of this absence in the educational standards is the recent American Chiropractic Association’s re-branding initiative that involved an extensive internal review by a consultant which found that the chiropractic profession is now very insular and has a wide variance in quality and treatment options for patients [25]. The American Chiropractic Association solution was to ask members to increase collaboration with other health care professionals and become more evidence-based. While laudable, it may be simplistic to expect that a profession can change quickly in this regard as such change is likely to be slow to happen and difficult to implement [26]. In order to obtain change, the target group should be as geographically local as possible, value diverse evidence and involve the use of multimethod programs [27]. This suggests that changes are best initiated at the undergraduate level and accreditation processes may be one such lever.

Future iterations of accreditation standards should consider the implementation of a more rigorous analysis of the available evidence and other health professions’ accreditation standards, as well as employing ‘outside’ appropriately qualified experts as mentioned above. Also recommended is the field-testing of new standards in order to make necessary ‘adjustments’ in positive directions possible within the chiropractic profession.

Accreditation standards require a common understandable and unifying language [28]. Previous studies have shown that follow-up analysis based on monitoring is required to ensure the language employed in any new standards is properly interpreted and that its impact is as intended [12]. Too often improvement has been assumed and not measured [1]. The new accreditation standards are considerably more detailed than the preceding standards. Nevertheless, the 2016 standards appeared to discuss approximately the same number of domains as the 2010 version but each in more detail. By being “wordier” this may address a concern that minimalistic language inhibits the interpretation and uptake of accreditation standards [29]. To this end, a revisit to all the stakeholders, especially CPs, for feedback on interpretation and implementation of the 2016 standards may provide valuable information for the CCE-International for continued improvement.

#### Overview and foreword

The 2010 standards concept of a chiropractor moved from giving particular attention to the relationship between the structural and neurological aspects of the body in health and disease to become a health profession concerned with the diagnosis, treatment and prevention of mechanical disorders of the musculoskeletal system in the 2016 standards. While there is confusion among chiropractors as to their scope of practice [30] patients do not suffer this quandary [31]. Patients want a practitioner who deals with musculoskeletal issues [32] and not wellness care or any type of primary prevention of musculoskeletal or public health-related disorders [33]. Thus, the more recent concept is likely to be in closer accord with patient expectations and the known evidence for the outcomes of manual therapy for musculoskeletal injuries.

Research suggests that a hybrid accreditation model involving regulatory compliance alongside quality improvement, such as the 2016 CCE-International standards, is continuing to evolve internationally and appears to be effectively promoting minimum standards and results in enhanced safety cultures [20, 34]. Consequently, we recommend accurate monitoring of the hybrid model with the intention of integrating this into future accreditation standards.

While there is some accreditation processes that have promoted the concept of “excellence” in chiropractic education more needs to be done. With few exceptions accreditation has largely focused on a pass-or-fail adequacy evaluation mechanism. Although in some instances a quality improvement standard has been added [35], a true excellence standard has not been introduced. Medical education regulators have taken steps to create an additional level of attainment to evaluate whether medical schools are capable of going above and beyond the traditional scope of accreditation by providing a superior level of education [36–38]. The intent of this is to recognize and promote outstanding performance of medical schools and provide role models for other medical educators. Many medical programs have engaged in this process and sought such recognition [39]. The CCE-International is a suitable vehicle to create a program such as this to incentivize and recognize quality chiropractic education.

#### Program/educational standards

The wider health community expects that accreditation assessment should incorporate the widespread use of objective educational outcomes measures [3, 40, 41]. The 2016 CCE-International standards, when compared to those of 2010, demonstrate alignment with this expectation. This is clearly stated in the Introduction of the 2016 standards and the increased frequency of related words further entrench this change in assessment of CPs.

There have been concerns over the lack of quality measures available to regulators for assessments of some of the stipulated standards [42]. For example, how is the requirement to create lifelong learners measured? The 2016 standards do not define in detail outcome measures or indicators that should be used for this purpose. CCEs could assist CPs by clearly stating which measures are best utilized to demonstrate achievement of the desired competencies. If none exists, or the quality is poor, then CCEs, CPs, and the profession at large could support research to this end.

Further, the frequency of the term “evidence-based” has been shown to be an indicator of the quality of accreditation standards and their regulation [15, 43]. The words ‘evidence-based’ neither appear in 2010 nor the 2016 standards. In fact the Glossary of the 2016 standards contains information explaining why the term is deliberately excluded. The nearest term is ‘evidence-informed’, which occurs once and in relation to student clinic patient care. Concerns have been raised about the failure of the chiropractic profession to embrace evidence-based practice and that the use of ‘evidence-informed’ is a form of soft resistance to the more widely accepted term evidence-based practice. There is a contention that the “evidence informed” practice places emphasis on practice experience and not on research [44]. In combination this indicates an apparent reluctance to align with accepted mainstream evidence-based health care education standards. A PUBMED search for “Evidence-informed practice” results in 123 articles, whereas “Evidence-based practice” results in 17,737. This speaks for itself as to the acceptance and common use of these terms.

The move toward less prescriptive faculty requirements and the removal of minimal standards for academic and clinical faculty in CPs may be viewed as further evidence of this reluctance. Likewise is the removal of the requirements for students to be able to critically appraise clinical studies, and scientific and clinical knowledge. Despite these limitations staff are expected to facilitate research to contribute to the chiropractic profession. Without prior knowledge on how to critically appraise research projects and research publications, it would be difficult for students and staff uneducated in research methodology, to absorb the full value of such activities.

Medical education views members of faculty as exemplars in the delivery of safe, effective, systems-based approaches to patient care, with the intention and ability of instilling ideas of quality values in the students they teach [45]. Faculty are expected to recommend the use of integrative approaches, inter-professional team-based patient-centred care that uses evidence-based medicine to provide safe and effective treatments for people in pain [46]. It is reasonable to assume that members of chiropractic faculty have an equally important role in the students they teach. However, it is difficult to see how the removal of minimal qualifications for faculty and the lack of evidence-based drivers are supportive of this concept. The reinstatement of these omissions from the 2010 standards is recommended as a starting point.

#### Procedures for initial accreditation and reaccreditation

Several studies have shown the benefits of accreditation standards that are collaborative and involve an inclusive process [12, 24]. The 2016 standards contain several words indicative of a trend toward a more collaborative approach. For example, the word “stakeholder” is considerably more frequent than before. This is congruent with studies showing that public/stakeholder involvement in the design and implementation enhances accreditation standards [24, 47]. Another way of enhancing the engagement and confidence of stakeholders is to adopt a policy of transparency in the accreditation processes [23, 47, 48].

However, some have suggested that transparency of the entire process reduces open communication between health educational programs and regulatory bodies [49] and is only acted on by a small number of citizens [50, 51]. Others have suggested it increases standards by contributing to consumer empowerment and affecting compliance through concern over public image [52]. Initial glances of CCE websites show varying levels of transparency with the CCE-Europe publishing site evaluation reports and the remaining choosing not to. There is no evidence to suggest that there have been adverse outcomes in chiropractic standards in Europe with the adoption of this standard. A recent systematic review of public health policy and practice found that ‘transparency’ is now considered a main moral value and a norm [21]. The authors can see no reason why all CCE standards should not reflect this societal norm. Further, the standards should not aim at protecting the schools (from insight) but to protect the public (from substandard education and hence from unsuitable clinicians).

Accreditation is predicated upon the reliability of site visitation teams’ judgments but the reliability of this process is unknown and difficult to study [49]. Consistent site team reports are more likely when reliability of the process and consistent application of standards are pursued [49]. A starting point would be to ensure that site team members are appropriately qualified, trained, instructed and provided with instruction manuals. No such requirement is found in the 2016 standards. International standards for medical school inspectors expect the team member to have extensive experience in the profession, with a minimum of experience in high managerial positions (ranging from 2 to 5 years), and profession-specific certification [53]. Some selection processes for medical inspectors have been known to incorporate lists of clearly defined personal attributes and competence such as communication, perceptiveness and administrative skills [53]. No research could be found identifying any aspect of site visitation of CPs. This would appear to be an area that requires further investigation to ascertain what best facilitates the uptake of accreditation standards and quality improvement among the CPs.

Graduates from for-profit colleges earn less than those from not-for profit colleges in the USA. For-profit colleges also tend to incur higher fees [54] and have higher student attrition rates [55]. Consequently, it is not surprising that concerns have been raised as to whether students can earn enough to justify the investment and pay back their student loans [56]. The omission of the requirement for the CP to be linked to a not-for-profit educational institution is therefore interesting. However, the 2016 standards appear to ensure that potential students have complete and objective information about the costs and expected benefits of a CP. This may also address the concern of aggressive and potentially misleading recruitment practices, poor ethical practices, and inappropriate commercial influences occurring in CPs, which have been documented in other health education programs [54, 57].

### What is not in the CCE-international 2010 and 2016 standards

This comparative study has only included data within the standards of CCE-International 2010 and 2016 standards. Relevant material may not be present in either version. One such area is the inclusion into chiropractic curriculums of non-evidence based constructs such as *subluxation* as an ‘objective’ lesion and *vitalism* as a model of treatment other than as a historical concept [15, 58]. This could be viewed analogously with an accredited Astronomy program that also teaches Astrology throughout its curriculum or an Ophthalmology program that includes Iris Diagnosis. Silence in CCE documents about such ‘sentinel’ terms could be interpreted as consent even though this may not be the intention.

This is particularly relevant as unorthodox chiropractic practice patterns, such as considering the chiropractic subluxation an encumbrance to the expression of health, anti-vaccination attitudes, and low levels of inter-professional referrals have been related to specific CPs suggesting that they are still actively teaching *vitalism* [8]. There is contemporary evidence that shows this occurs in some chiropractic institutions, even after having passed through a CCE inspection and being granted re-accreditation. For example “LIFE’s (Marietta GA campus) educational and clinical philosophy is based on Vitalism. .” [59]. Also, the New Zealand College of Chiropractic states on its web-page “The philosophy of chiropractic is vitalistic in that it acknowledges the body’s ability to self-regulate, coordinate and heal. This philosophy guides our curriculum, strategy and culture throughout the College” [60]. To our knowledge, both colleges have been CCE accredited.

A further example is the inappropriate use of the term ‘subluxation’ in CPs apart from its use as an historical term. A previous study has counted the number of courses mentioning ‘subluxation’ in North American CPs. It found the Palmer College (Florida campus) devoted 22% of its curriculum to courses mentioning ‘subluxation’ followed by Life University (Marietta GA campus 16%) and Sherman College (13%) [61].

We recognise that regulation is more than ‘rule compliance’ and should encompass methods and mechanisms that encourage CPs to go beyond mere compliance [36, 37, 62]. However, at this point in time, some CPs are not actively pursuing the mainstream healthcare norm which is evidence-based practice. Silence in accreditation documentation on such matters hinders the integration of chiropractic into the wider healthcare community. What is required are prescriptive standards that are clearly evidence-based, actively monitored and enforced.

### Recommendations

This review has sought to identify similarities and differences between the CCE-International 2016 and 2010 accreditation / educational standards that has led to the identification of a number of issues. Based on these, we make some recommendations that are summarised in Table 4. If these recommendations were adopted, then outcomes, such as a uniform and high standard of accreditation standards based on evidence and shown to be effective before implementation, would be more likely to be similar across all CCE-controlled regions. This could assist in ensuring and safeguarding the international trust in CPs’ ability to produce practitioners who can deliver ethical, safe, and quality care across international borders. It would also likely assist chiropractors becoming accepted by other health care professions.

**Table 4:** Summary table of recommendations

	Recommendations In relation to Standards	Justifications
1	All participants in the accreditation process and their qualifications for the task are clearly stated. A broad range of participants including health consumers and non-chiropractic educators should be included.	To ensure the construction of accreditation standards are transparent and draw on as wide a range of expertise as possible.
2	A review of the evidence-base of the CCE-International accreditation/educational standards	This would allow stronger alignment with contemporary medical standards and increase acceptance of chiropractic into the mainstream health care system.
3	A trial methodology of the new standards.	The CCE-International could address potentially problematic areas such as poor comprehension, compliance or uptake.
4	Adoption of industry standards of ‘qualifications’ for faculty and site investigation team members (as well as appropriate training).	Enhanced CP teaching and research with improved faculty qualifications. Increased quality of site visitation members offers more expertise for quality improvement, and evaluations that are more efficient and effective.
5	Transparency of accreditation processes e.g., publication of (re) accreditation reports and recommendations.	CPs are mindful of public image and marketability and this would reinforce compliance with standards. Increases consumer empowerment.
6	Regular reviews and integration of emerging research to continually update accreditation standards. Especially with respect to quantifying required CP outcome measures.	More efficient and accurate assessments of CPs.
7	The adoption of an evidence-based approach to all aspects of the teaching and practice of musculoskeletal healthcare.	This is the expectation of society, patients and health care education in general.
8	Create an award system as part of chiropractic accreditation for excellence in education.	To incentivize chiropractic programs to create high quality education and desirable models for other CPs to emulate.

We recognise that there is a substantive cost in engaging experts to assist with accreditation, establishing an awards system, conducting an evidence-based review of accreditation standards, trialling them with quality research and publishing the findings in the peer-reviewed literature. Debate exists in the medical education literature over who should shoulder this financial impost [63]. Such a debate will need to take place for chiropractic education with attention to how such funding can take place without compromising the independence and integrity of the CCE-International.

### Methodological considerations

This was a comprehensive comparison that included all the material from the 2010 and 2016 CCE-International Accreditation Standards. The screening method matched all the 2010 areas and subareas to the 2016 standards. The authors remain confident that they have found the areas, subareas and terms and that they appropriately classified them for accurate comparisons. The search for other key terms, however, could perhaps have resulted in other findings and conclusions.

There was a high degree of agreement between the two readers / authors on text interpretation and allocation. Hence, there was not a need to draw on the third author for any interpretations that could not readily be resolved by discussion.

Thematic identification can result in data being interpreted several ways and it is difficult to know if the themes identified are relevant [16]. We have made the judgements for theme identification clear and there was good agreement across coders. Nevertheless, we may not have identified every relevant word. However, we are confident in the findings, as the authors have published in the area, have worked with CCEs and, consequently, are familiar with similar documentation. Finally, the adjectival word list was large and this also reduces the likelihood of omitting many important words.

It should also be borne in mind that the frequency of terms does not necessarily relate to the quality of the document and we recognize that program evaluation extends beyond these documents alone and requires an extensive self-evaluation, inspection and review process. However, the contents of these standards are clearly the foundation for such evaluations, and are therefore important documents to scrutinize.

## Conclusions

This comparison of the old and the revised CCE-International accreditation standards revealed that the new standards are more detailed when describing the competencies required for the graduating chiropractor and the re-accreditation process for CPs. On the positive side, it also shows that progress is being made aligning with current research and accepted standards. Interestingly, these standards are now based on a definition of the chiropractic profession dealing with musculoskeletal problems and apparently not opening the door to the treatment of other diseases via the spine.

However, there is still considerable progress to be made with respect to the rigour of the application of an evidence-based approach to accreditation standard development and trialling the standards before implementation. The term ‘evidence-based’ is still lacking. We hope that this is not an attempt to amalgamate the two large factions within the profession, i.e. those inclined towards vitalism and those who are more interested in treating musculoskeletal problems. Full transparency of the expertise, qualifications and affiliations of all participants and stakeholders would allay such concerns.

We noted the removal of minimal qualifications for faculty, that it is no longer necessary for the CP to belong to a not-for-profit educational institution, and we noted also the absence of specified qualifications for site visitation teams.

An opportunity exists to further improve the CCE-International standards with the addition of standards specifically addressing known non-evidence based curricula as well as producing desired models of education with the creation of an awards scheme for recognition of excellence.

## Abbreviations

CCE: Council on Chiropractic Education
CCE-International: Council on Chiropractic Education – International
CP: Chiropractic Program
